# Echolocation intensity and directionality of perching and flying fringe-lipped bats, *Trachops cirrhosus* (Phyllostomidae)

**DOI:** 10.3389/fphys.2013.00143

**Published:** 2013-06-28

**Authors:** Annemarie Surlykke, Lasse Jakobsen, Elisabeth K. V. Kalko, Rachel A. Page

**Affiliations:** ^1^Institute of Biology, University of Southern Denmark, Odense M.Fyn, Denmark; ^2^Institute of Experimental Ecology, University of UlmUlm, Germany; ^3^Smithsonian Tropical Research InstituteBalboa, Ancón, Panamá

**Keywords:** echolocation, directionality, intensity, sonar beam, perch hunting

## Abstract

The Neotropical frog-eating bat, *Trachops cirrhosus*, primarily hunts stationary prey, either by gleaning on the wing, or in a sit-and-wait mode hanging from a perch. It listens passively for prey-generated sounds, but uses echolocation in all stages of the hunt. Like other bats in the family Phyllostomidae, *T. cirrhosus* has a conspicuous nose leaf, hypothesized to direct and focus echolocation calls emitted from the nostrils. *T. cirrhosus* is highly flexible in its cognitive abilities and its use of sensory strategies for prey detection. Additionally, *T. cirrhosus* has been observed to echolocate both with closed and open mouth. We hypothesize that its flexibility extends to echolocation call design. We investigated the effect of hunting mode, perching or flying, as well as the effect of mouth opening, on the acoustic parameters and directionality of the echolocation call. We used a multi-microphone array, a high-speed video camera, and a microphone-diode-video system to directly visualize the echolocation sound beam synchronized with the bat's behavior. We found that *T. cirrhosus* emits a highly directional sound beam with half amplitude angle (HAM) of 12–18° and DI (directionality index) of ~17 dB, among the most directional bat sonar beams measured to date. The directionality was high both when flying and when perching. The emitted intensity was low, around 88 dB SPL at 10 cm from the mouth, when hanging, but higher, around 100 dB SPL at 10 cm, when flying or just before take-off. Our data suggests that the limited search volume of *T. cirrhosus* sonar beam defined by the high directionality and the rather low intensity of its echolocation calls is adapted to the highly cluttered hunting habitat and to the perch hunting mode.

## Introduction

Echolocation is one of the key adaptations enabling the successful and rapid radiation of bats. Bats emit high frequency signals and use the returning echoes to orientate in darkness, to detect and localize prey, and to find roosts. There is considerable variation in echolocation call design across bat species, and a large number of studies have shown that within species, individuals can flexibly adapt the time- and frequency features of their echolocation calls to the situation and task at hand (e.g., Neuweiler, 1989; Schnitzler and Kalko, 2001). Recent data demonstrate that this flexibility also extends to the intensity (Brinkløv et al., 2010) and directionality of the sonar signal (Surlykke et al., 2009b; Jakobsen et al., 2013). Intensity and directionality are critical in defining the sonar search volume, i.e., the volume of space ahead of the bat in which objects are ensonified with sufficient sound energy to reflect detectable echoes. Some bats hunt in a sit-and-wait hunting mode, hanging from a perch and scanning the surroundings for potential prey by rotating the head and body. Perch hunting is often seen in rhinolophid bats (Neuweiler et al., 1987; Jones and Rayner, 1989), but also in other families, e.g., Phyllostomidae (Weinbeer and Kalko, 2004), Megadermatidae (Audet et al., 1991), and Nycteridae (Fenton et al., 1987). Because echolocation call production can be coupled with wing beats, it may require close to no extra energy to produce echolocation sounds in flight (Speakman and Racey, 1991). However, overall flight costs are high and perch hunting is far less costly energeti-cally than continuous flight (Voigt et al., 2010). It is, however, unknown whether perch hunting poses special constraints on the echolocation, thus, promoting adaptive changes to intensity and directionality as well as other acoustic features of the echolocation calls.

The fringe-lipped bat, *Trachops cirrhosus* (Phyllostomidae), occurs in the Neotropics, from southern Mexico to southern Brazil (Reid, 1997). It roosts in hollow trees and flies a short distance (1–2 km) to its foraging grounds, where it gleans prey over a relatively small area (3–4 ha) (Kalko et al., 1999). *T. cirrhosus* uses both continuous flight and perch hunting when foraging. Radio-telemetry studies found that *T. cirrhosus* makes long flights (>2 min) early in the evening, hunting on the wing along streams and over ponds, likely predominantly for frogs, i.e., túngara frog [*Engystomops* (formerly *Physalaemus*) *pustulosus*]. Later in the night, when frog calling activity decreases, *T. cirrhosus* switches to perch hunting, sallying from a perch in short flights of less than 1 min, presumably hunting insect prey, such as forest katydids (Kalko et al., 1999). *T. cirrhosus* relies primarily on prey-emitted acoustic cues to detect and localize its prey, and can use species-specific frog mating calls to assess prey palatability (Tuttle and Ryan, 1981). It has been suggested that *T. cirrhosus* can detect the loud, conspicuous calls of túngara frogs and other preferred frog species even while on the wing, but when listening for katydid wing beat or landing sounds, or eavesdropping on their faint, high frequency and often low duty cycle calling song, a hang-and-wait strategy is more effective (Kalko et al., 1999).

Eavesdropping on prey-generated acoustic cues has been well documented in *T. cirrhosus*, both in field and flight cage experiments (Ryan et al., 1982). However, even though *T. cirrhosus* primarily uses passive listening to detect and localize its prey, it produces echolocation calls throughout the hunting approach (Barclay et al., 1981). Flight cage experiments show that it can use echolocation and spatial memory (Page and Ryan, 2008) to detect prey that falls silent upon approach, and can use both echolocation and chemical cues in the final stages of prey assessment (Page et al., 2012). *T. cirrhosus* emits typical phyllostomid calls, consisting of short, multiharmonic sweeps of low intensity. In confined space, such as the laboratory, most energy is in the third (sweeping from 110 kHz down to 80 kHz) and fourth harmonic and call duration is less than 1 ms. The cruising pulse rate is around 25 Hz, but in the final phase before attacking their prey the rate increases to around 80 Hz (Barclay et al., 1981).

A member of the phyllostomid family of leaf-nosed bats, *T. cirrhosus* has a prominent nose leaf, extending from the base of the nostrils. Nose leaves of phyllostomids are fairly similar in overall shape but differ greatly in size (Vanderelst et al., 2010). It is generally accepted that phyllostomids emit echolocation calls through the nostrils. In all probability, the nose leaf, which is not found in mouth-emitting bat families like e.g., Vespertilionidae or Emballonuridae, has a role in shaping and steering the sonar sound beam (Hartley and Suthers, 1987; Vanderelst et al., 2010). However, directionality has rarely been measured directly, and new data from flying *Carollia perspicilliata* (Brinkløv et al., 2011) demonstrated a narrower sonar beam when flying than earlier data from sitting bats had indicated (Hartley and Suthers, 1987). Thus, phyllostomid bats, like vespertilionids, may have the ability to flexibly modify their beam shapes to adapt to a given situation (Surlykke et al., 2009b; Jakobsen and Surlykke, 2010; Jakobsen et al., 2013). Since *Trachops* hunts while hanging from a perch as well as on the wing, it offers an excellent opportunity to study whether sonar search volume (intensity and directionality) is adapted to hunting strategy. In addition to listening for the sounds of its prey, we also frequently observed that *T. cirrhosus* opened its mouth while echolocating from a perch. Several other phyllostomid species have also been observed to open the mouth while echolocating (Tschapka, page 11 in LaVal and Rodriìguez-H, 2002), which might influence the sound emission by changing the emission site or altering the head-related transfer function. Thus, a second purpose of this study was to determine if *T. cirrhosus* adds an extra level to its sonar flexibility by being able to echolocate both through the nostrils and through the open mouth.

## Materials and methods

### Animals

**Figure 1:**
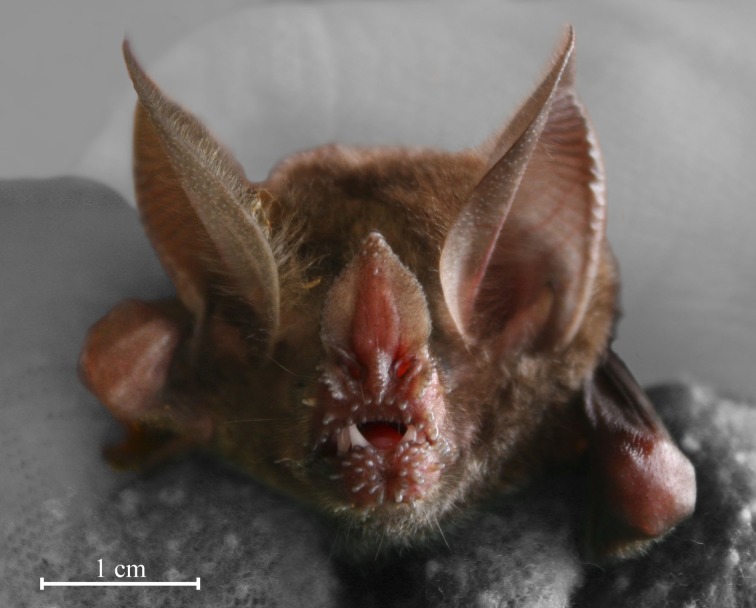
***Trachops cirrhosus* has a nose leaf with a lancet that is 9 mm from tip to nostrils**. The large ears and the characteristic tubercles around the chin and lips are also clearly visible.

**Figure 2:**
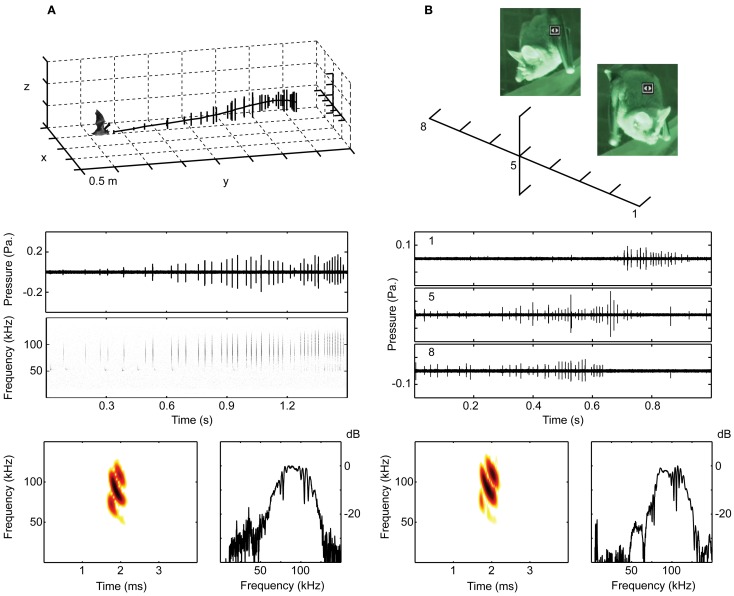
**The bats were recorded with microphone arrays when hanging from a perch or when flying. (A)** Shows the set-up in 2012 with 11 microphones in a cross-shaped array, 7 horizontal and 3 above and 1 below the center microphone. The middle panel shows oscillograms and spectrograms of the echolocation calls emitted in the flight illustrated in the upper panel, where each vertical line is a call. The lower panel shows a spectrogram and a spectrum of one of the calls from the same sequence. **(B)** Shows a recording from 2008 of a perching bat. The 10 microphone array had 8 microphones on a horizontal line at the height of the bat's mouth. The middle panel shows oscillograms of a single echolocation sequence, recorded simultaneously on microphones 1, 5 and 8. Stills from the infrared video illustrate the bat turning its head from right to left. Due to the high directionality of the sonar beam, the calls are only visible on the channels at which the bat is aiming, i.e., channel 8 when the bat is facing right in the beginning and channel 1 when the bat is facing left in the end of the trial. Below are shown spectrum and spectrogram of one of the calls from this sequence.

We captured bats on Barro Colorado Island, in Soberanía National Park and the areas surrounding Gamboa, Panama, using mist nets set along small streams and ponds at dusk. We recorded echolocation calls from 6 *T. cirrhosus* with a mean capture weight of 32.5 g (range 28–37 g) and mean forearm length of 58.8 mm (range 57.2–60.7 mm). We measured the length of the lancet of the nose leaf for 9 other individuals (Figure 1) from the tip of the nose leaf to the center of a line connecting the two nostril centers (mean ± SEM: 9.2 ± 0.3 mm), as measured in Brinkløv et al. (2011). In 2006, we recorded from two females, in 2008, from two males, and in 2012, from two males. All bats were held and tested in screened, outdoor flight cages. Bats from 2006 and 2008 were tested in a 4.5 × 4 × 2.5 m flight cage on Barro Colorado Island; bats from 2012 were tested in a 5 × 5 × 2.5 m flight cage in Gamboa. We recorded the bats in two behavioral situations: (1) while they were hanging from their perch, a short branch in the upper corner of the flight cage ca. 1.75 m above the floor, scanning the environment by turning head and body, and (2) while they were flying toward a loudspeaker placed on the floor of the flight cage ca. 2.5 m horizontal distance from the perch (Figure 2).

### Sound recordings

We recorded all bats with arrays of ¼″ (G.R.A.S) microphones (without grids) amplified by G.R.A.S. 12AA or Avisoft ¼″ power modules. We digitized the signals at 250, 500, or 300 kHz per channel using either an IOTech Wavebook or an Avisoft USGH (Avisoft Bioacoustics, Berlin, Germany), and stored data on a laptop computer. In 2006, we used four microphones in a T-shaped array with approximately 30 cm distance between microphones placed above the loudspeaker on the floor. In 2008, we used 10 microphones with ca. 30 cm distance, 8 G.R.A.S. ¼″ on a linear horizontal line and an Avisoft condenser microphone (CM16) above and below the 5th microphone, which was 90 cm in front of the bat and at the same height as its mouth. In 2012, we used 11 ¼″ G.R.A.S. microphones with 25 cm distance in a cross shaped array, 7 horizontally and 3 above and one below the center (4th) microphone. The array was ca. 5 m from the wall on which the bats perched (Figure 2).

### Video recordings

All trials were conducted in red (25W red light bulb) and infrared light (Wisecomm IR045 LED and Conrad infrared spot) to minimize the bat's use of vision. All trials were video-recorded. In 2006, we used a Sony nightshot DCR-SR45 camcorder. Bats were presented with speakers broadcasting the calls of túngara frogs, a preferred prey species, and rewarded with a prey item on the speaker. In 2008, we recorded bats hanging from a perch with two Sony nightshot DCR-SR45 camcorders. One video focused on the bat's head and the other on an array of diodes connected directly via custom build amplifiers to a second 4 × 4 array of microphones (Knowles) spaced by 46 cm horizontally and 26 cm vertically. The minimum distance to the bat was 1 m translating into a resolution of around 20–30°. The diodes had 16 steps of light from green over yellow to red over a 30 dB range, and were adjusted to just emit green light at background noise. Thus, the diodes corresponding to the microphone(s) ensonified by the bat would emit orange or red light according to the sound level on the microphone. We combined the footage from the two camcorders into one movie using an Extron PIP 422 Picture-in-Picture Processor thus giving us on-line synchronized feedback simultaneously about the bat's head and nose leaf position, mouth opening, and sonar beam aim. In 2008, we additionally recorded the perching bats with high-speed video (CamRecord 600, Optronis, Germany) at 500 frames per second. The high-speed video was synchronized to an Avisoft Ultrasound recording system using an Avisoft condenser microphone (CM16, Avisoft Bioacoustics, Berlin, Germany) and a one-channel Avisoft USG digitizer. The high speed video and synchronized sound was stored on a laptop computer. Both video systems were used to find sequences where the mouth was clearly open, and the diodes were used to control that the sonar beam hit the diodes in the directions the head and nose leaf was aiming.

In 2012, we recorded sounds from two bats, both when they were perching and when they were flying toward a speaker broadcasting frog calls for a food reward. In contrast to the previous recordings, the bats would perch at a relatively random location on the cage wall and thus approach the speaker from a variation of directions. The bats were video recorded with a Sony HDR-CX550V camcorder, which was adjusted to the bat's position before each trial.

### Analyses

We estimated source levels and directionality in flight for the two bats recorded in 2006 and the two bats recorded in 2012. We obtained the bats' flight paths using the arrival time differences at the microphones to localize the bats at the time of each echolocation emission. We determined source levels and directionality only from calls where the beam aim was in the center of the microphone array. Because of the array configuration we obtained only horizontal directionality from the bats recorded in 2006 and 2008, but both directions for the 2012 data. Using the estimated positions, we calculated source levels (emitted intensity referenced to 10 cm from the bat's mouth measured in dB SPL rms) by adding transmission loss (geometric spreading loss and atmospheric attenuation) and microphone directionality (Brüel and Kjær, 1982) using the method described in Jakobsen et al. (2012).

We estimated source levels and directionality for perching bats with either open or closed mouth for the two bats recorded in 2008 and for the two bats recorded in 2012. In 2008, the array allowed for determinations of the horizontal directionality, but only indications of beam aim in the vertical plane. We used the camcorder video combined with the diode display to guide us to sequences, where the bat's mouth was either clearly open, or clearly closed, and analysed the acoustic behavior in detail by using the high speed video and ¼″ microphone recordings.

**Figure 3:**
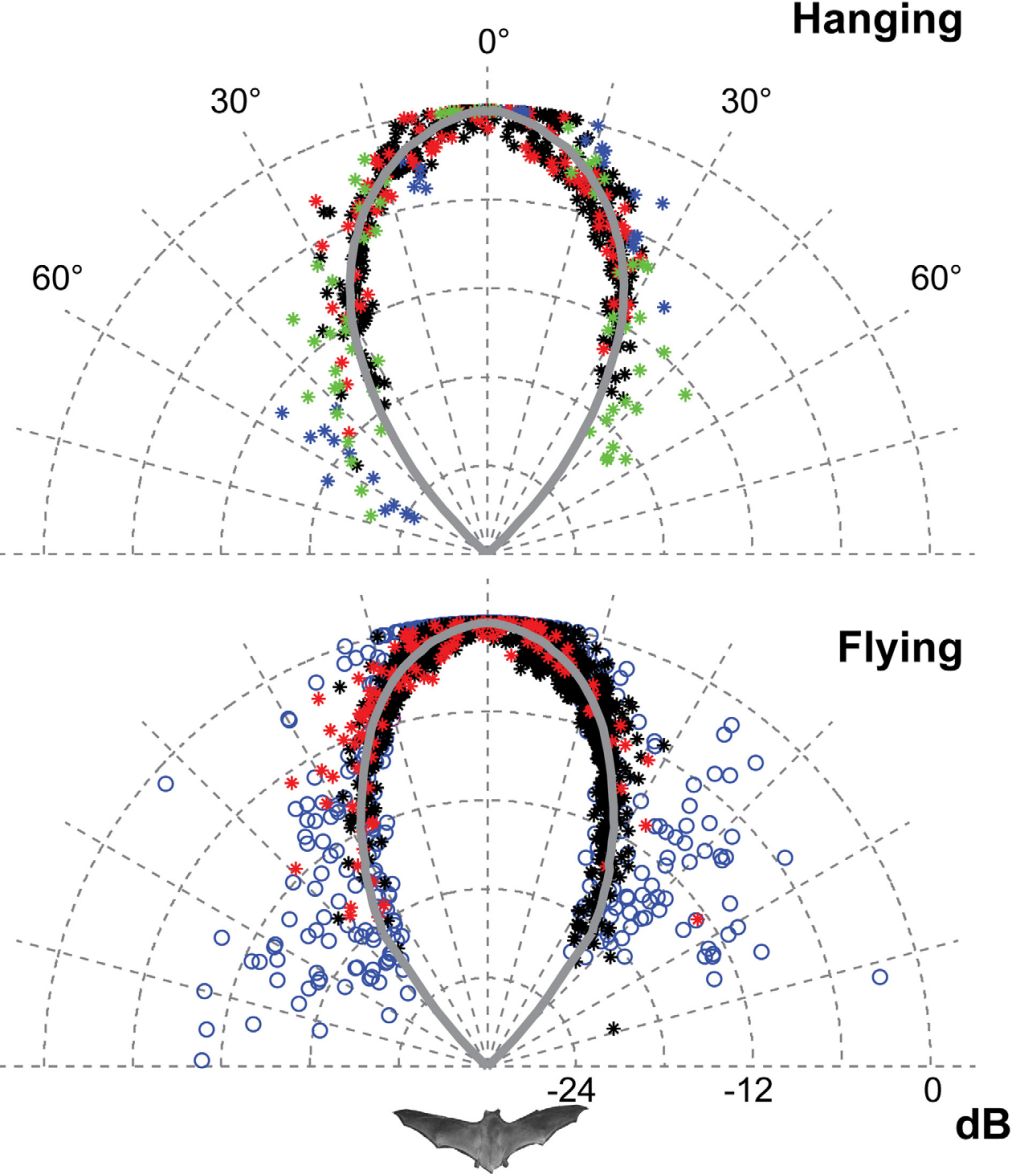
**The horizontal directionality when flying and hanging**. The data for hanging bats are from 2012 (red and black) and from 2008 (blue and green). The data for flying bats are from 2012 (red and black) and from 2006 (blue). Each point is the normalised amplitude in that direction. The curves are the average of the measured values pooled in 1° bins and extrapolated using a fitted second order polynomial. Only data from 2012 was used for the traces and DI estimates because of the higher degree of control of beam aim. The sonar beam is very directional, both when flying and hanging. HAM was 18°.

**Figure 4:**
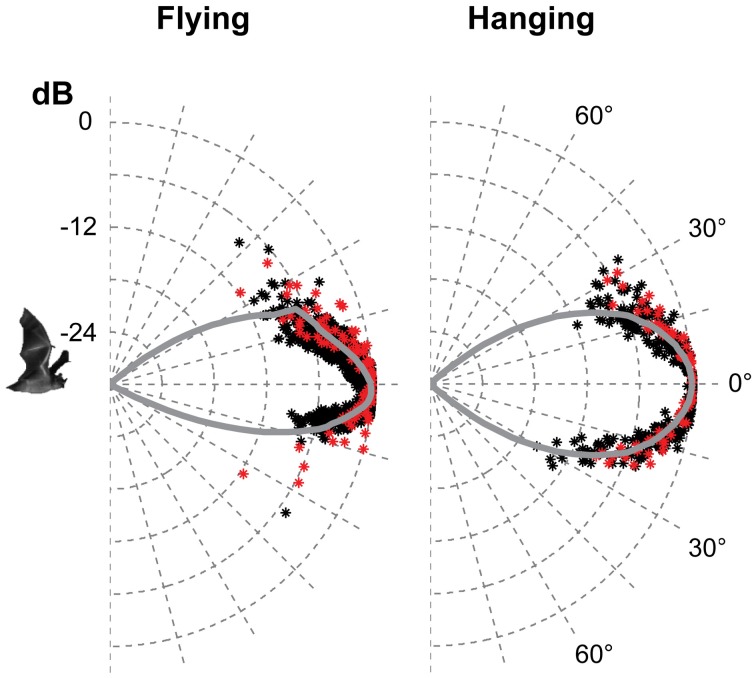
**The vertical directionality when flying and hanging**. The data are from the two bats recorded in 2012, red for one bat and black for the other. Each point is the normalised amplitude in that direction. The curves are the average of the measured values pooled in 1° bins and extrapolated using a fitted second order polynomial. The beam was symmetrical in the vertical direction when hanging with HAM of 18°. When flying, the beam was more directional than when hanging, and asymmetrical with HAM in the upwards direction of 15° and 12° in the downward direction.

Directionality can be quantified as half amplitude angle (HAM) or directivity index (DI). HAM is the off-axis angle, where the amplitude of the signal has declined by 6 dB. DI compares on-axis sound pressure with the sound pressure of an omnidirectional emitter producing a signal of equal energy. For all estimates of source level and directionality, we estimated beam-aim by fitting a 2nd order polynomial to the recorded beam pattern, using the peak of the polynomial as a proxy for beam aim. For details see Brinkløv et al. (2011). We calculated the DI as in Møhl et al. (2003):

$$DI=10\log_{10}\left(\frac{2}{\sum_{i=1}^{N}\left(b_{i}\times \sin\left(\nu_{i}\right)\times \Delta \nu \right)}\right)$$

where *b*_*i*_ is the *i'*th sample of an interpolation of the measured beam pattern, ν_*i*_ is the angle, between 0 and π radians, and Δν is the angular interval between interpolation points. *N* is the number of samples. The expression assumes rotational symmetry. To obtain the interpolation of the measured beam pattern, we pooled the measured relative sound pressures (both horizontal and vertical) into 1° bins and averaged them. We then extrapolated the measurements to obtain the complete sound field around the bat by fitting a second order polynomial to the average of the measured beams (Figures 3, 4).

## Results

### Echolocation sounds when flying and hanging from a perch

When leaving the perch and flying toward the loudspeaker the bats always echolocated and we never observed opening of the mouth. The echolocation calls had main energy in the third harmonic with *F*_peak_ (the frequency with maximum energy) at ca. 90 kHz, and less energy in the second and fourth harmonic (*F*_peak_ at 60 and 120 kHz, respectively). The calls were short, between 0.3 and 0.9 ms, and repeated with irregular pulse intervals of 30–120 ms when the bat was far away from the loudspeaker. At closer range the sonar pulses were grouped with increasing pulse number and decreasing pulse interval within the groups, which were 70–100 ms long, consisting of 3–10 pulses. Within groups the pulse interval was relatively constant (Figure 2A).

Since the source level decreased as the bat approached the loudspeaker and simultaneously got closer to the floor, we calculated source levels from calls emitted, when the bats were still far enough from the microphone array. At this distance we also got good S/N on all four or all eleven microphones. The maximum source levels (referenced to 10 cm) were recorded immediately after the bat left the perch and were 103 ± 3 dB, and 99 ± 4 dB for the two bats in 2006 and 102 ± 3 dB and 99 ± 3 dB for the two bats in 2012.

When on the perch, the bats often hung silently for long periods, but they echolocated when they lifted their heads and started scanning the surroundings, turning the head and the whole body while rapidly moving the pinnae of the ears back and forth. A typical emission pattern is illustrated in Figure 2B. Bats emitted trains of pulses with pulse intervals ranging from 20 to 120 ms, often 30–50 ms. The pulse duration was the same as when flying, i.e., around 0.5–0.8 ms and again the main energy was in the third harmonic with *F*_peak_ at 85–90 kHz. The apparent amplitude modulations (Figure 2B, middle panel) are not due to changes in emitted sound level, but reflect the bats' rapid scanning movements combined with the directionality of the calls. From the video we determined approximate scanning angles. The body turned ca. 45° from extreme to extreme, in addition the head turned an extra ca. 45°, thus totaling ca. 90° turn of head aim angle. While perching the source level was 86 dB ± 10 dB SPL and 88 dB ± 7 dB SPL for the two bats recorded in 2008. Right before taking off from the perch they emitted more intense calls, with source levels ca. 10 dB louder: 99.7 ± 3.4 dB for the two 2008 bats, demonstrating that they control the emitted amplitude over a large dynamic range. In 2012, the source levels when the bats were perching were estimated to be higher, 102 ± 2 dB and 98 ± 4 dB SPL. The difference is likely to be caused by the much longer distance from the hanging bat to the microphone array in 2012 (Figure 2), only allowing for recording of the loudest calls directed toward the array.

### Directionality of the echolocation sounds

We determined the directionality in three different situations: (i) flying, (ii) hanging from the perch with closed mouth, and (iii) hanging with open mouth. We never observed any of the six bats flying with open mouth, but video from 2008 showed several sequences where the bats had open mouth while echolocating from the perch (Figure 2B).

Only the horizontal directionality could be extracted from the 2006 and 2008 data, but both vertical and horizontal directionality were determined from the 2012 data (Figures 3 and 4 red and black data points). Estimates of beam shapes and statistical analyses were performed on the 2012 data, where we recorded with many microphones, but the values for 2008 confirm the measurements and are plotted in the same graphs (Figure 3 blue and green data points). The beam was narrow with a horizontal HAM of 18°, both when flying and hanging (Figure 3). In the vertical direction the measured directionality of the sonar beam was slightly narrower when the bats were flying than when hanging. HAM was ca. 18° both up and down when hanging, but when flying HAM in the upward direction was 15°, and only 12° in the downward direction (Figure 4). DI for the combined data-set, was 16 dB when hanging and 17 dB when flying. When all data from both scenarios were pooled, DI was 17 dB. DI estimated using only the vertical directionality data was 17 dB for hanging bats and slightly more directional, 19 dB, for flying bats. Hence, the data indicated a narrower and more asymmetrical beam when the bats were in flight (Figure 4) although the differences between directionality from hanging and flying bats were not statistical significant.We regressed angle (absolute value) against sound pressure (Pa) for each bat's echolocation calls produced while flying and while hanging and found no difference in the slope of these two lines for either bat (two- and one-tailed tests for difference between two population regression coefficients, *P* > 0.05 for all).

**Figure 5:**
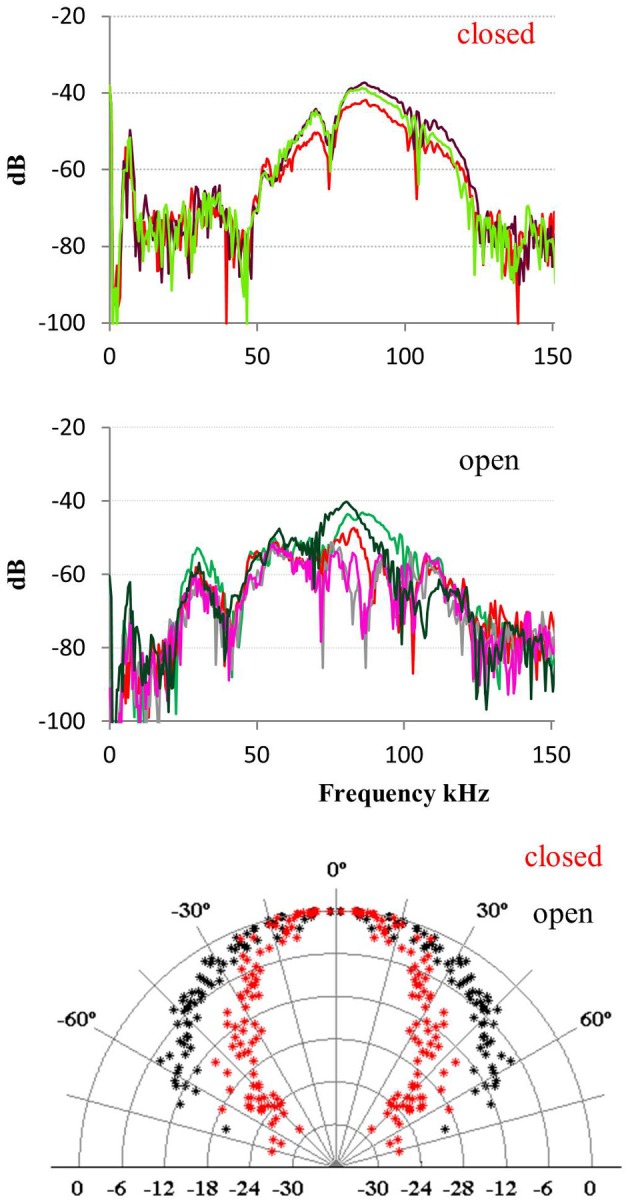
**Calls emitted with closed and open mouth**. When one of the bats recorded in 2008 emitted calls with closed mouth (upper panel) it had most energy in the third harmonic (*F*_peak_ 90 kHz) and some in the second and fourth harmonic. When it emitted calls with open mouth (middle panel) it also emitted substantial energy in the first harmonic (*F*_peak_ 30 kHz). The effect of this was that the bat added a component with broad directionality (lower panel, black data points) when it called with the mouth open, compared to the directionality calculated for closed mouth calls with most energy around 90 kHz (red points).

The data from bats hanging and echolocating with open mouth is somewhat inconsistent. One of the two bats recorded in 2008 emitted calls with open mouth that were indistinguishable from the calls emitted with closed mouth, for all acoustic parameters measured: spectrum, amplitude, and directionality. In contrast, when the other 2008 bat emitted calls with open mouth, there was an additional pronounced peak in the spectrum around the first harmonic (the fundamental) at 30 kHz, which was not seen when this bat echolocated with the mouth closed. The directionality at 30 kHz was as expected much broader than at 90 kHz with HAM of 45° (Figure 5). We did not have synchronized video documentation in 2012 to allow us to know exactly when the bats had open mouth, but we did not record any signals with a prominent fundamental from either of the two, neither when hanging nor when flying.

## Discussion

The recordings from all six *T. cirrhosus* showed typical phyllostomid echolocation calls, i.e., short, multiharmonic calls with most energy at high frequencies, around 90 kHz, in the third harmonic, and often of relatively low output intensity. Barclay et al. (1981) reported a peak frequency closer to 75 kHz. However, distance, off-axis recordings, as well as microphone directionality will all low-pass filter the sounds. Here we report spectral characteristics of calls recorded on-axis and compensated for those low-pass effects, implying that the emitted *F*_peak_ is really around 90 kHz. The calls are very directional both while flying (DI = 17 dB) and while perching (DI = 16 dB).

The narrow sonar beam of *T. cirrhosus* corroborates data from *Carollia perspicillata,* (HAM 16° horizontally and 14° vertically and DI = 17 dB, calculated from the original data) the only other phyllostomid species for which directionality has been measured in freely flying bats (Brinkløv et al., 2011). *C. perspicillata* is somewhat smaller than *T. cirrhosus* (41–45 mm vs. 57–65 mm forearm length and ~18 g vs. ~30 g) but the lancet of the nose leaf is almost the same size (8 mm vs. 9 mm) (Brinkløv et al., 2011). Both bat species emit very similar echolocation signals, with *F*_peak_ around 90 kHz. Measurements from anesthetized *C. perspicillata* (Hartley and Suthers, 1987) as well as modeled directionality from *Phyllostomus discolor* (Vanderelst et al., 2010) demonstrate that the high directionality in the vertical plane is due to the extended nose leaf whereas the two nostrils determine directionality in the horizontal plane. Presumably this holds for *T. cirrhosus* too, since the overall shape and size of the nose leaf are quite similar in all three species. Given the similarity in nose leaf morphology and echolocation call features it is not surprising that the sonar beam directionality is similar in *C. perspicillata* and *T. cirrhosus*. Still, it is important to note that the nose leaf is not the sound emitter, but instead likely functions as a baffle, and thus its exact size is not expected to affect the sound field as much and as predictably as the size of the emitter. In mouth-emitting bats the emitter size appears to be the gape size and thus mouth-emitting bats presumably have more mechanical control over directionality (Jakobsen et al., 2013) than nostril-emitting bats. However, we are still far from understanding the functional significance of motor control of nose leaf shape for sonar directionality (Hartley and Suthers, 1987; Feng et al., 2012). Vanderelst et al. (2010)'s model predicted a sonar beam that was symmetrical in the horizontal direction, but asymmetrical in the vertical direction, with a main lobe, which was wider above than below the acoustic axis. This is in accordance with our results for flying *T. cirrhosus,* whereas we found the vertical directionality to be symmetrical for hanging bats. The difference in sonar beam shape between hanging and flying bats was not statistically significant, probably due to the limited number of bats and data. Potentially, it indicates active control of the beam by bending the nose leaf in the vertical direction as has also been suggested for another phyllostomid bat, *Macrophyllum macrophyllum* (Weinbeer and Kalko, 2007), but further investigations are needed to clarify. If motor control of the nose leaf functions in dynamic active adaptation of the sonar beam axis and directionality in phyllostomid bat, in particular in flight, this once more emphasizes the importance of verifying models and measurements based on static morphological data with measurements from live naturally behaving bats.

Our data does not provide a clear conclusion to whether mouth opening is part of beam control. The data on one bat very clearly showed addition of lower frequency and thus a broader component of the beam, but data from only one bat is far from conclusive. If more data should show this to be of functional significance, it would add yet another level of flexible control of sonar search volume in *T. cirrhosus* or perhaps more generally in phyllostomids that open the mouth while echolocating.

While our data was not sufficient to show a significant difference between flying and hanging, it did show unequivocally that the beam is very narrow under all circumstances, similar to the beam of flying *Carollia perspicillata*. Although *T. cirrhosus* is carnivorous and *C. perspicillata* is frugivorous, they both take predominantly stationary prey in dense clutter, so in some respects their foraging ecology and demands on their echolocation systems are quite similar. An advantage of a very narrow beam is that it provides inherent directional information: if the energy is focused in a narrow angle around the axis of the sound beam, off axis objects will only be ensonified with low intensity sound and their echoes will be much reduced, leaving salient echo information to come from the direction of the sonar beam axis. Narrow beams thus also reduce the load on the processing system. Interestingly, the opposite adaptation is seen in vespertilionid bats, which broaden the beam in confined space (Surlykke et al., 2009b; Jakobsen et al., 2013). This difference might reflect that we have not yet understood the function of directionality. However, it might also be evidence of the enormous diversity of echolocating bats. Bats of different families have different strategies for detecting insects close to background vegetation: bats that use frequency modulated echolocation calls (FM bats) shorten the calls in closed habitats to make discrimination easier along the time axis, whereas bats that produce constant frequency calls (CF bats) produce extremely long narrow banded calls to discriminate between prey and background along the frequency axis (Schnitzler, 1967; Neuweiler, 1989; Moss et al., 2011). Along the same lines, we hypothesize that bats, depending on their phylogeny, hunting habitat, and prey type, use different strategies to deal with clutter. Phyllostomid bats hunting stationary prey may benefit from a narrow beam to decrease the load on the processing system and focus on the important target, whereas vespertilionid bats hunting primarily moving prey may broaden the beam to prevent the prey from escaping out of the echolocation beam (Goerlitz et al., 2010; Jakobsen and Surlykke, 2010) and also to “keep an eye” on the clutter in order not to collide while pursuing erratic prey.

Radio-tracking studies suggest that *T. cirrhosus* often switches from gleaning to perch-hunting (Kalko et al., 1999). Perch hunting is thought to reduce the energy consumption compared to constant flight (Neuweiler et al., 1987; Voigt et al., 2010). Another advantage of perch hunting is the possibility of using a wider search angle when hunting prey. Our data indicates a wide search angle (ca. 90°) for *T. cirrhosus* although not quite as wide as the 200° estimated for rhinolophid bats (Neuweiler et al., 1987). In addition, scanning may reduce clutter. Bats sample their environment sequentially (Surlykke et al., 2009a), and when scanning perch hunting bats sequentially ensonify objects within a wide angle of directions. By integrating the input over time, they can create an auditory scene in great detail with much less off-axis clutter than a broader beam covering the same total angle would provide.

Finally, an underappreciated advantage of perch hunting might be an improved signal-to-noise ratio, since there is no wind noise from flight. Wind noise has never been measured for flying bats, but has been estimated to increase detection thresholds from the standard mammalian threshold of 0 dB SPL to around 20 dB SPL (Surlykke and Kalko, 2008). Stationary bats with large ears have been shown to have minimum hearing thresholds below 0 dB SPL (down to −20 dB SPL) (Long, 1977; Hoffmann et al., 2008). When stationary, big ears not only function as large acoustic antennae, but by their independent movements, also provide directional information by differentiating and focusing incoming acoustic input. In flight, in contrast, big ears are likely to create even more noise due to their higher air resistance. If we assume a source level according to our data of around 100 dB SPL at 10 cm (rms) and an increase in detection threshold from 0 to 20 dB SPL when flying compared to hanging (conservative estimate given the large ears of *T. cirrhosus*, Figure 1), we can estimate detection ranges for insect-sized prey with a target strength of −20 dB (Surlykke et al., 1999) using the simple form of the sonar equation:

EL=SL−2TL+TS

EL = echo threshold level, TL = one way transmission loss (geometric spreading and atmospheric attenuation at 90 kHz, 28°C, 80% relative humidity), TS = target strength (Surlykke and Kalko, 2008). A perching bat would be able to detect insect echoes at a distance of 2.9 m, but only at 1.6 m when flying. The bat can lower its source level by up to 20 dB when hanging without paying with detection range compared to when flying. In fact, at 88 dB SPL, the echolocation source level we mostly recorded on the perch, the detection distance would be ca. 2.1 m, i.e., substantially longer than when emitting 100 dB SPL in flight. Thus, in addition to reducing energy consumption from flight, perching may create even larger acoustic advantages from reduced noise and more precise directional information for big-eared than for other bats.

In conclusion, our results show that *T. cirrhosus* emits a very narrow sonar beam both when hanging and flying. To understand the functional and ecological significance of different hunting modes it is important to integrate all aspects of hunting behavior, not only energy consumption, but also the critical features of echolocation including intensity and directionality. Our study indicates that the high directionality and moderate sound level in phyllostomid bats are adapted to the mode of hunting, i.e., largely motionless prey in dense clutter, and is not governed or affected by additional sensory cues the bats may receive from their quarry (e.g., passive acoustics, olfactory cues, etc.).

### Conflict of interest statement

The authors declare that the research was conducted in the absence of any commercial or financial relationships that could be construed as a potential conflict of interest.
